# Supplementary material for: Are carbon emissions trading and green financial instruments synergistic? -Comprehensive quantitative research based on content analysis
Source: PLoS One. 2024 Mar 7;19(3):e0298601. doi: 10.1371/journal.pone.0298601 (PMC10919601; doi:10.1371/journal.pone.0298601)
Supplement: S1 Data — (ZIP) [file pone.0298601.s001.zip › Data/地级市Z_F工作报告.zip!/╕ú╜¿/╚¬╓▌╩╨/└┤╫╘╣1⁄2╓┌║┼í╛┬φ┐╦╩2╛▌═°í┐_2005.docx]

各位代表：

　　 现在，我代表泉州市人民政府向大会作工作报告，请各位代表连同《泉州市国民经济和社会发展第十一个五年规划纲要（草案）》一并审议，并请政协委员和其他列席人员提出意见。

　　一、“十五”经济社会发展回顾

　　“十五”时期是泉州建设海峡西岸经济区现代化工贸港口城市的重要时期。全市认真贯彻落实党的十六大精神，始终坚持以邓小平理论和“三个代表”重要思想为指导，树立和落实科学发展观，牢牢抓住发展第一要务，弘扬创新、创业、创造精神，认真开展保持共产党员先进性教育活动，扎实推进全面建设小康社会，主动融入海峡西岸经济区建设大局，战胜了前进中的各种困难，保持了经济社会发展的好势头、安定稳定的好局面。经济结构进一步优化，经济增长质量和效益稳步提高，全市生产总值保持福建省前列，财政收入大幅增加；经济体制改革不断深化，市场经济体制不断完善；开放型经济格局进一步形成，利用外资和对外贸易取得新突破；固定资产投资较快增长，基础设施建设取得新成就，城市发展和城镇化进程步伐加快；“三农”工作取得新成效；城乡最低生活保障制度全面推行，社会保障体系逐步建立；社会主义民主法制建设和精神文明建设继续加强，各项社会事业协调发展，全市基本实现宽裕型小康目标。市十三届人大一次会议确定的“十五”计划主要目标任务提前完成。初步统计2005年全市生产总值1804亿元（按经普结果调整的数据待以后公布），比2000年增长80.8％；人均生产总值2930美元，比2000年增加1007美元；三次产业比例由2000年的7.7∶53.5∶38.8调整为2005年的5.4∶54.1∶40.5；财政总收入153.36亿元，其中一般预算收入76.11亿元，“十五”期间年均分别增长21.7％和21.7％；城镇居民人均可支配收入14209元，农民人均纯收入6123元。“十五”期间，全市累计出口105亿美元（海关口径），比“九五”翻一番；实际利用外资49亿美元，比“九五”增长41.9％；全社会固定资产投资1522.4亿元，是“九五”的1.71倍；中心城市建成区面积从2000年的40平方公里扩大至70平方公里；城镇化率从2000年的39％提高到49％。荣获中国优秀旅游城市、国家卫生城市、国家园林城市、国际花园城市、最佳中国魅力城市、中国品牌经济城市、全国法制宣传教育先进城市、全国创建文明城市工作先进城市等称号，实现全国社会治安综合治理优秀地市“二连冠”、全国民族团结进步模范集体“三连冠”、全国科技进步先进市“五连冠”、全国双拥模范城“五连冠”。

　　2005年是“十五”最后一年，我们积极构建民营经济、开放创新、城市发展、先进文化和和谐社会五大跃升平台，经济社会持续快速健康发展。晋江、南安、惠安、石狮、安溪等5县（市）进入第五届全国县域经济基本竞争力百强县（市）。所有县（市）均保持全省经济实力十强或经济发展十佳。一年来，主要工作和成效是：

　　致力构建民营经济跃升平台，产业集聚效应日益显现。推进“228”产业集群规划，完成10个产业集群规划及若干行业专项规划。深入实施项目带动战略，83个市在建重点项目完成投资81亿元，建成南埔电厂一期、石狮服装城等一批项目，动工建设炼化一体化、泉州船厂等一批项目。突出抓好42家省重点企业、100家市级重点工业企业及4147家规模以上企业的跟踪服务。出台实施建设节约型社会工作意见和促进工业用地集约利用的意见，完成80个循环经济示范项目，建成标准厂房面积34.67万平方米。举办泉州市企业劳务合作洽谈会和第三届银企资金供需洽谈会，帮助企业解决用工和资金短缺等困难问题。深入实施名牌战略，新增中国驰名商标16件、中国名牌产品18项、国家免检产品38项、中国出口名牌8个、出口免验企业1家。

　　致力构建开放创新跃升平台，经济发展动力持续增强。深化行政审批、国有企业、投融资体制改革。成立市城市管理行政执法局，市、区管理体制改革迈出重要步伐。新增股份公司3家、境外上市企业3家。创新招商引资机制，全年实际利用外资12亿美元（报表口径）。积极开拓国际市场，出口商品总值达32.07亿美元（海关口径）。深入推进泉港合作八大平台、泉澳四项合作，成功举办“2005泉州？香港投资项目推介洽谈会”。泉台交流与合作进一步加强。泉州国家级出口加工区获准设立。

　　致力构建城市发展跃升平台，港城联动战略全面启动。沿海大通道全线基本贯通，城建“一二三四”项目顺利推进，“一场一馆”、泉三高速公路（泉州段）、桥南片区等项目动工；市区一、二环路规划方案基本确定，“三片三带”和“四规四改”工作进展顺利。完成泉州港口总体规划修编，泉州港秀涂3万吨级多用途码头起步工程、斗尾30万吨级原油码头等深水码头建设步伐加快。全年港口货物吞吐量4046万吨、集装箱吞吐量63万标箱。城市经济加快发展，招商银行、民生银行在泉州设立分行；沃尔玛、SM、来雅等大型商贸企业落户泉州。

　　致力构建先进文化跃升平台，社会各项事业健康发展。组织实施市级以上科技计划项目和重大科技专项220项，新增省级高新技术企业25家。加快中国闽台缘博物馆等19个重点社会事业项目建设。巩固提高城乡义务教育成果，改造中小学危房27万平方米，妥善解决12.74万名进城务工人员子女就近入学，对1.77万名农村贫困家庭学生实行“两免一补”。完成市级及5个县（市、区）疾病控制与卫生监督体制改革，有效控制霍乱疫情的流行。文化、体育工作取得新进展。全市人口与计划生育工作取得新成效，鲤城、丰泽、永春3个县（区），洛江、石狮、惠安、德化、晋江5个县（市、区），泉港、南安、安溪3个县（市、区）分别被评为全省计生一、二、三类先进县（市、区）。社会科学、文学艺术、新闻出版、广播电视、民族宗教、外事侨务、口岸查验、妇女儿童、老龄、残疾人、气象、防震减灾、档案、地方志等工作取得新成绩，人民防空、国防动员、民兵、预备役和双拥工作再上新台阶。

　　致力构建和谐社会跃升平台，城乡居民生活质量进一步提高。出台实施加强农业农村工作的若干意见，市本级农业类支出比经常性财力增长高出3.6个百分点。“六千”水利工程、金鸡拦河闸重建等水利重点工程建设取得进展，“358”水库除险保安工程基本完成。齐心协力抵御台风袭击，严防严控禽流感疫情。实施“造福工程”搬迁2950人，转移农村劳动力5.13万人。新增4个县（市、区）、34个乡镇、377个村实现宽裕型小康。大力创建国家环保模范城市，改善人居环境，晋江、洛阳江流域水环境保护与污染控制投入加大，基本实现市十三届人大一次会议2号议案决议的目标任务。进一步巩固发展社会保险和城乡低保成果。深化“平安泉州”创建活动，实现“一升一降”目标。完成安全生产控制目标任务。此外，出台建设公共服务型政府的实施意见，大力加强政府自身建设，落实党风廉政建设责任制，政府行政能力和工作效能得到提高。

　　各位代表，“十五”期间，我市经济建设和社会各项事业取得了辉煌的成就，这是全市人民在市委领导下，团结奋进、艰苦拼搏、扎实工作、积极奉献的结果。在此，我代表市人民政府向全市广大干部群众，各民主党派、工商联和无党派人士，驻泉省部属单位，驻泉部队、武警官兵，向为泉州建设作出贡献的外来员工，向所有关心、支持和参与泉州现代化建设的台港澳同胞、海外侨胞、海内外朋友致以崇高的敬意！对人大代表、政协委员和全市人民给予政府的高度信任和大力支持表示衷心的感谢！

　　回顾5年来的工作实践，我们的做法和体会是：

　　一是坚持融入大局，明确目标定位。5年来，我们围绕建设海峡西岸经济区，认真贯彻福建省新一轮发展“四个推进”、“四个基本”、“四个关键”等战略部署，结合泉州实际，大力推进现代化工贸港口城市建设。特别是在发展县域经济、壮大中心城市、提升民营经济、加快体制创新等方面，大胆实践，敢于突破，取得了明显的成效。“十五”时期，成为泉州经济社会发展加快、人民得到较多实惠的时期。

　　二是坚持“两个发展”，增强综合实力。5年来，我们坚持加快发展、科学发展相统一，紧紧扭住发展第一要务不动摇。在发展过程中，更加注重在调整优化结构和转变增长方式上下功夫，在有效节约、合理配置资源和保护生态环境上下功夫，在依靠科技进步和提高劳动者素质上下功夫。坚持“民营与外资、轻工与重化两条腿走路”，坚持“生产性、商贸性项目先上，关系国计民生的基础设施及配套项目先上”，加快石油化工、修船造船、汽车制造、电子信息等新兴产业发展，增强经济持续发展的后劲。

　　三是坚持改革开放，优化发展环境。5年来，我们坚持向深化改革要办法，靠体制创新找出路，从扩大开放寻活力。加大能源、交通等建设投入，提高基础设施承载力；努力营造良好的“大通关”环境，有为应对反倾销和技术性贸易壁垒，增强贸易争端预警和处置能力；从建立健全制度、完善机制体制入手，深入开展“优化环境、优质服务”活动，努力形成“想干事、能干事、干成事”的导向和氛围，全市干部群众应对挑战、攻坚克难、谋求发展的意识持续增强。

　　四是坚持统筹兼顾，促进和谐发展。5年来，我们注意处理好改革发展稳定的关系，处理好发展过程中经济与社会、人与自然、城市与乡村、沿海与山区的关系，着力解决不协调、不平衡问题，促进经济社会生态全面、协调、可持续发展。重视“三农”工作，更加关心困难群体，努力改善“老、少、边、岛”群众的生产和生活条件；重视“平安泉州”建设，大力推进社会治安综合治理，不断完善社会应急机制、社会纠纷调处和利益协调机制；重视人的全面发展，不断提高城乡文明程度，促进社会公平公正、安定有序、和谐发展。

　　五是坚持项目带动，落实工作责任。5年来，我们敢于探索和创新，把实施项目带动战略作为全市经济社会发展的重要抓手，建立健全项目的生成、培育、服务、责任机制，以项目提升优势、落实服务、检验成效、推动发展。特别是实施“五个一”工作机制，实现了创新项目管理方式新突破。一批事关全局和长远的大项目、好项目投建，促进了经济活力的激发、生产要素的集聚、工作责任的落实和发展合力的形成。

　　六是坚持执政为民，解决热点难点问题。5年来，我们坚持把群众冷暖安危挂在心上，让发展成果更多更普遍地惠及百姓。全市累计减轻农民负担15.45亿元，清理拖欠农民工工资2.12亿元；完成为民办实事项目55件105项，认真办理人大代表议案建议和政协提案5813件；办理群众信访件35988件；办理房屋所有权证108656件；高度重视防灾减灾和公共突发性事件监测预警体系建设，投入7.78亿元完善中心市区排洪排涝系统，万众一心抗击“非典”取得重大胜利；努力解决与人民群众生活息息相关的收入、就业、价格、住房、公共卫生、食品安全、社会保障等问题，海内外泉州人创新、创业、创造的凝聚力进一步提升。

　　当前，我市经济社会发展中也存在不少困难与问题，主要体现在：城乡差距仍然较大，农村医疗卫生、文化教育等社会事业发展相对滞后，“三农”工作有待进一步加强。产业结构调整和经济增长方式转变任务艰巨。企业自主创新能力较弱，规模企业、龙头企业带动作用不强。资源集约利用与综合开发水平不高，土地闲置、水资源污染与浪费较为突出。国际贸易摩擦增多，原材料涨价、融资困难压力加大，熟练技工、高技能人才缺乏，体制、要素、人才对经济增长的瓶颈约束凸显。中心城市规模偏小，集聚辐射带动功能亟待提升。城市化、工业化进程中被征地农民养老、转岗就业压力较大。安全生产基础比较薄弱，社会治安不容乐观。部分公务人员服务意识、法制意识、责任意识不够强，个别部门工作效率低下，个别公务人员行政不作为、乱作为现象时有发生，投资软环境建设仍是人民群众普遍关注的焦点。此外，全市仍有部分工作未达到预期目标，主要是：第三产业比重距“十五”目标尚差2.5个百分点；2005年“两会”期间人大代表和政协委员集中反映的31项建议中还有2项仍在实施中；2005年度确定的190个政府工作分解项目尚有25个正在抓紧推进。对此，我们要增强忧患意识和责任感，采取更有力措施，认真加以解决。

　　二、“十一五”时期主要目标任务

　　“十一五”时期是我市发展的重要战略机遇期。和平、发展、合作成为时代潮流，经济全球化、区域一体化深入发展，科技创新和产业转移步伐加快，为我市全面参与国际分工与合作创造了有利环境。全国区域发展新格局形成，中央支持海峡西岸建设，福建的发展站在一个新的历史起点上，为我市进一步拓宽发展空间提供了有利条件和现实途径。同时也必须清醒地看到，我市面临的国际国内竞争日益激烈，加快发展面临较大压力。我们必须抢抓机遇，增强使命感和紧迫感，高起点谋划新一轮发展的目标任务，推动经济社会转入科学发展的良性轨道，开创全面建设小康社会新局面。

　　“十一五”时期我市经济社会发展的指导思想是：以邓小平理论和“三个代表”重要思想为指导，深入贯彻党的十六届五中全会、省委七届十次全会和市委九届十二次全会精神，以科学发展观统领经济社会发展全局，以构建和谐社会为战略任务，认真落实省委“四个推进”、“四个基本”、“四个关键”等战略部署，坚持加快发展、科学发展，积极拓展“五大发展空间”，全力推进现代化工贸港口城市建设，构建充满活力的创业型城市，在海峡西岸经济区建设中走在前列，基本实现全面建设小康社会目标。主要奋斗目标是：基本实现全面建设小康社会目标，2010年全市生产总值达3100亿元以上，人均生产总值达4800美元，财政总收入超过320亿元，力争突破350亿元，农民人均纯收入和城镇居民人均可支配收入分别达8500元和19200元，单位生产总值能耗低于全省平均水平，城乡之间、区域之间协调发展，社会事业全面进步，社会稳定和谐；基本形成大城市框架，框架内城市建成区面积180平方公里，人口180万人，城镇化率达60％；基本建成全省乃至全国重要的先进制造业基地和物流中心，石油化工、纺织服装产值超1000亿元，建材、鞋业产值超500亿元；基本建成亿吨大港，成为我国东南沿海重要港口；基本建成文化旅游强市，增强文化产业和旅游业的竞争力和影响力，争创全国文明城市、中国最佳旅游城市。为此，要抓好以下几方面工作。

　　建设社会主义新农村。按照“生产发展、生活宽裕、乡风文明、村容整洁、管理民主”要求，坚持“多予少取放活”和“工业反哺农业、城市支持农村”方针，加快推进新农村建设。大力发展现代农业，推进农业产业化经营，提高农业综合生产能力。深化农村改革，根据自愿、有偿原则，依法流转土地承包经营权，健全被征地农民的合理补偿和社会保障机制，基本完成乡镇机构、农村义务教育和县乡财政管理体制等改革任务。扩大农村公共服务，加大对农村基础设施、防灾减灾和社会事业投入，基本建立新型农村合作医疗制度。加强村镇规划建设，整治村容村貌，改善乡村生产生活环境。加强农村基层自治组织建设，规范村务公开、民主管理制度。培养有文化、懂技术、会经营的新型农民。加速农村劳动力转移，加大扶贫开发力度，健全减轻农民负担监督机制，千方百计增加农民收入。

　　全面提升产业竞争力。坚持走新型工业化道路，发展壮大五大新兴产业，加快建设海峡西岸重要的石油化工、船舶修造、汽车制造、电子信息、生物医药基地。改造提升五大传统产业，建成我国重要的纺织鞋服、建筑建材基地和海峡西岸重要的机械制造、工艺制品、食品饮料基地。重视发展海洋经济，重点发展临港工业、港口物流、滨海旅游、远洋运输和海洋生物医药产业。不断增强产业集聚功能，以产业园区、产业基地和项目建设为载体，做大做强“228”产业集群。大力发展现代服务业，培育壮大旅游业、现代物流业，加快金融保险、电子商务、房地产、信息咨询等服务业发展。

　　建设自主创新型城市。整合科技资源，加强五大国家级科技示范工程建设，建立健全区域自主创新体系。充分发挥企业在技术创新中的主体作用，支持企业建立研发机构。加强产学研合作，加快科技成果转化和产业化。做好全国知识产权试点城市工作。引导企业采用国际标准，鼓励企业参与制定国家标准，扶持企业创建名牌、品牌。培育一批具有自主知识产权、自主品牌、核心竞争力强的大企业。加大科技投入，增强全市原始创新、集成创新和引进消化吸收再创新能力。

　　促进区域协调发展。围绕建设海峡西岸经济区现代化工贸港口城市，科学布局五大经济区域。发展壮大中心城市，全面完成城建“一二三四”项目；整合城市资源，适时调整行政区划，推进中心城市规划区建设；发挥中心城市龙头带动作用，优先发展公共交通，加快供水、供电、供气及污水处理、垃圾无害化处理等市政设施建设。大力发展区域经济，沿海县（市）重点发展临港工业、特色产业和配套产业；山区县大力发展特色产业、资源加工业和生态型产业。加快城镇化步伐，制定落实鼓励城镇加快发展的政策措施，有序引导农民进入城镇就业。推进港城联动，重点建设泉州湾中心港区和湄洲湾南岸泉州新港区，实现亿吨大港目标。加强基础设施建设，完善海、陆、空立体交通网络，加强能源建设，健全城乡防灾减灾体系。

　　激发体制创新活力。深化国企改革，完善国有资产监管运营机制。健全公共财政体制，优化支出结构，防范政府债务风险。全面推进企业投资项目核准制和备案制，实行政府投资决策责任制度。推进农村信用社改革，创新信用担保方式。壮大民营经济，建立健全市场准入、税收管理、金融贷款、信用评价等服务体系。继续整顿规范市场经济秩序，加快社会信用体系建设。规范发展要素市场，健全现代市场体系，发展壮大资本市场，扩大直接融资比重。继续推进行业协会、同业公会改革与发展。

　　提高对外开放水平。加快经济国际化进程，大力吸收发达国家和地区的跨国公司和大财团投资，鼓励设立生产基地、研发中心、采购中心，引导外资投向基础设施、高新技术产业、装备制造业、现代服务业，全面提高利用外资水平。转变外贸增长方式，实现从供货大市向出口大市转变。实施“走出去”战略，支持有比较优势的企业到境外直接投资，推动一批企业到境外上市融资。加强与台港澳侨合作与交流。发挥“五缘”优势，拓展“六求”作为，力争在承接台湾石化、电子、机械、现代服务业等产业转移上有较大突破，扩大对台航运和文化科技交流。深化泉港合作八大平台建设和泉澳四项合作。做好新形势下的侨务工作。加强区域协作，主动对接“长三角”、“珠三角”，密切与内陆地区经济联系，延伸我市发展腹地。

　　建设资源节约型和环境友好型社会。认真实施建设节约型社会工作意见，全面推进节能节水节材节地，大力发展循环经济，合理利用自然资源，开发推广资源节约的替代技术，力争“十一五”末期基本形成节约型增长方式和消费模式。加强生态环境建设，降低污染物排放总量，环境综合评价指数进入全省前列。实施晋江水资源节约配置、保护利用规划，健全生态补偿机制，继续抓好“两江”流域综合整治，加强生态脆弱区治理。推进城乡绿化工作，保护公益林和湿地，构筑生态安全屏障，努力实现人与自然的和谐发展。

　　构建以人为本的和谐泉州。推进和谐社会建设，健全党委领导、政府负责、社会协同、公众参与的社会管理格局。推进人才强市，加强党政人才、企业经营管理人才、专业技术人才、高技能人才和农村实用人才队伍建设。实施科教兴市，坚持教育优先，整合各类教育资源，全面实施素质教育，基本普及高中阶段教育，大力发展职业教育，提高高等教育水平，健全终身教育体系，构建学习型社会。重视发展文化事业和文化产业，完善城乡公共文化服务体系。大力弘扬闽南文化，服务祖国和平统一大业。加强城乡公共卫生和医疗服务，不断提高人民群众健康水平。办好2008年第六届全国农民运动会。进一步加强人口和计划生育工作，加快发展妇女儿童事业，积极应对人口老龄化。完善社会保障体系。加强安全生产和社会治安综合治理工作，健全社会稳定的预警体系和应急管理工作机制，建设“平安泉州”。

　　三、关于2006年政府工作主要安排

　　2006年是实施“十一五”规划的开局之年，各级政府各部门要按照市委、市政府统一部署，开拓进取，真抓实干，努力实现又快又好发展，确保“十一五”开好局、起好步。预期全市生产总值增长12.5％，财政一般预算收入增长17％，固定资产投资增长18％以上。经济社会其他各项指标也作了相应安排。重点要推进七个方面工作：

　　（一）加快推进城乡统筹，建设社会主义新农村

　　搞好总体规划。制定切实可行的实施方案，统筹谋划新农村建设的目标、政策、任务、步骤和项目。落实支农政策。加大对“三农”的支持力度，从项目、资金、政策上向安溪、永春、德化、洛江、泉港、南安的“老、少、边、岛”和贫困乡村倾斜。继续搞好扶贫开发，组织沿海县（市、区）与安溪、永春、德化开展帮扶“结对子”；实施“造福工程”，搬迁安置5000人。加快现代农业建设。围绕促进农业增效、农民增收，大力推广农业科技实用技术和优良品种，调整优化农产品种植结构，发展“两高一优”农业，努力提高龙眼、茶叶、花卉等大宗农产品的产业化水平。围绕“抓龙头、建基地、创品牌、拓市场”工作思路，实施“1333”工程。发展海洋养殖捕捞和海产品加工业，加快建设深沪、祥芝国家中心渔港。鼓励各地传统农产品和优势农产品申请注册地理标志证明商标或集体商标。深化集体林权制度改革，加强森林防火和植树造林，完成林业发展年度目标。加强农村公共服务。加大对农村交通、通讯、电力、自来水等基础设施的投建力度，加强农田水利基础设施建设，启动小山塘除险加固工程，完成金鸡拦河闸重建、南高干渠、晋江下游防洪岸线（金塔堤段）整治和“六千”水利工程年度建设任务。推广农村沼气工程建设。完成150个50户以上自然村“村村通”广播电视任务。按照“四改四化”要求，着力解决农村饮水、排水、厕所以及生产生活垃圾处理等问题。加强农村文化建设。新增教育、卫生、文化、计划生育等事业经费要向农村倾斜。加强农村文化阵地和载体建设，继续开展“三下乡”活动，丰富群众文化生活。实施“科技入户”、“农村支教”、“农村支卫”等工程。认真做好村委会换届选举工作，提高村民依法自治水平。深入开展农村精神文明建设，精心搞好农村保持共产党员先进性教育活动，为建设社会主义新农村提供组织保证和精神支持。

　　（二）加快推进项目带动，拓展产业发展新空间

　　强化项目带动。搞好“十五”和“十一五”有关重点项目建设安排的衔接，加快福厦铁路（泉州段）、泉三高速公路（泉州段）、走马埭围垦工程等一批重大续建工程项目建设。推进新建项目建设，搞好项目前期工作。举办第四届银企资金供需洽谈会，抓好第三届福建项目成果交易会签订的414个项目特别是10个投资超亿元大项目的跟踪落实。争取南埔电厂二期，泉州北、惠安500千伏变电站，江南、德化、长新等7个220千伏变电站和晋江液化天然气（LNG）电厂动工建设。壮大产业集群。组织实施“228”产业集群规划，增强产业配套能力。继续抓好100家市级重点工业企业、66个省级工业经济新增长点项目。落实省、市全面提升民营经济发展水平政策措施，帮助民营企业解决发展中的困难问题。引导建设和鼓励使用标准厂房，保障产业集群、重大项目用地。发展新兴产业。以福炼1200万吨／年炼油、80万吨／年乙烯一体化项目为龙头，重点发展化工原料、精细化工、三大合成材料及其后加工业；抓好中化1200万吨／年炼油首期工程500万吨／年重油加工、泉港重油催化热裂解、石狮60万吨／年PTA等项目的前期工作。以造船项目列入国家“十一五”船舶发展规划为契机，在加快修船项目建设的基础上，争取造船项目夏季投建。以中国一汽“解放”卡车项目投建为突破口，加快鲤城江南、丰泽城东等现有汽车配件产业资源整合，发展专用车、汽车零部件制造等项目。以福建光微的微波射频技术和移动通信直放机及功能模块为重点，推进国家微波通信特色产业基地建设。同时，加快培育发展生物医药产业。

　　（三）加快推进港城联动，构建城市跃升新平台

　　抓紧城乡规划编制。完成泉州市城市总体规划修编、市域城镇体系规划、都市区协调发展规划及“十一五”综合交通规划编制。开展城市公交、电力、绿化、环卫等专项规划修编，推进泉州石化基地、出口加工区、洛秀组团、城市商务区规划编制。加快村镇规划，完成10个乡镇、40个中心村的规划调整。抓紧城建项目建设。继续实施城建“一二三四”项目，确保序时进度。推进市行政管理服务中心、桥南片区、城东片区建设。抓好江南、见龙亭经济适用房项目。加快江南、洛秀、城东、东海、北峰组团、双阳片区供水管道铺设和旧城区供水排污管网改造。抓紧城市燃气工程建设，确保2007年供气。加快旧泉永路拓改、南立交桥、朋山岭第二隧道及延伸段等项目建设，改善城市交通环境。大力发展城市经济。千方百计扩充中心城市经济总量，突出南抓泉州出口加工区、北抓洛秀组团，开拓新的经济增长空间。积极发展现代物流业和服务业，编制实施现代服务业发展规划和港口物流业规划，建设物流园区，推进连锁经营、物流配送、电子商务，发展金融、保险、信息、法律服务、社区服务业。认真实施泉州市旅游发展总体规划，加快旅游资源整合，打造“海上丝路”、惠女风情、南少林等旅游品牌。推进港城联动。完成泉州港港区和重点作业区控制性详细规划修编。加快泉州湾中心港区、湄洲湾南岸泉州新港区和深沪湾、围头湾中小型港区规划建设，重点抓好斗尾30万吨级原油码头、泰山10万吨级石化码头及仓储工程等建设，促进中化集团物流、仓储码头落户泉州。做好泉州港航道疏浚工作。理顺港口管理体制，推进通关作业和查验监管模式改革创新。积极开辟近洋、内贸集装箱、液体化工等运输业务，争取实现港口年吞吐量4500万吨、集装箱80万标箱目标。

　　（四）加快推进改革开放，增创区域竞争新优势

　　增创体制改革新优势。深化行政管理体制改革，减少和规范行政审批。完善公共财政体制，狠抓财源建设和增收节支，坚持依法治税、依法理财。继续深化农村各项改革，稳妥推进农村税费、乡镇机构、农村义务教育和农村社会化服务体系改革。深化国有和集体企业改革，重点抓好省五建公司、市汽车运输总公司、省公路一公司等企业股份制改造。支持海天轻纺、先创电子等企业改制和冠福家用、浔兴拉链上市申报。增创外向带动新优势。鼓励具有自主知识产权、自主品牌的商品出口，提高加工贸易的产业层次。实施出口市场、出口商品和经营方式、经营主体多元化。密切关注当前纺织服装“特保”、技术贸易壁垒、反倾销等发展动态，增强贸易争端预警和处置能力。加快开拓国际市场，积极参与中国—东盟区域经济合作。创新招商引资工作机制，着力引进先进适用技术、现代化管理经验和专门人才，抓好资金和技术密集型项目、产业链和重要生产基地项目招商，鼓励现有外资企业增资扩营。全力推进泉州出口加工区建设，确保通过国家验收。增创台港澳侨新优势。主动承接台湾产业转移，特别是吸引台湾大企业来泉投资；抓住海峡两岸农业合作实验区扩大到全省的有利契机，深化泉台农业合作。办好海峡两岸农洽会、海博会。抓好申报国家级台商投资区的跟踪落实，做好对台直航基础工作，力争在物流、旅游、金融、服务等行业对台招商有新突破。扩大泉台经贸文化交流与合作，办好南安石井海峡农产品物流中心；以泉州港石井港区获准成为与金门直接往来客运口岸为契机，加快配套设施建设，尽快投入使用。全面推进泉港合作八大平台建设和泉澳四项合作。积极申报泉州晋江机场为对外开放口岸。扩大与海外侨亲和华人社团的联谊，做好侨务工作。

　　（五）加快推进科教兴市，实现各项社会事业新发展

　　加快科技创新。着力建设创新型城市，提高自主创新能力。大力推进行业研发中心、生产力促进中心、高新技术孵化中心、特色产业基地和博士后工作站等建设，开发推动产业升级的关键共性技术和配套技术，鼓励企业增加科研投入。市、县两级投入科技经费6000万元以上，组织实施200个科技项目和重大科技产业化项目。推进创建国家知识产权试点城市工作，加大知识产权保护力度。用好现有通信资源，建立泉州市中小企业信息化与技术服务应用平台，争创国家级企业工程技术中心。推动科技公共服务平台建设，加快自主创新的支撑体系建设。优先发展教育。深入实施教育现代化“八大工程”，提高各级各类教育发展水平。扎实推进“双高普九”。对农村义务教育阶段学生全部免收杂费，对困难家庭学生提供免费课本和寄宿生活费补助，依法保障进城务工人员子女接受义务教育。加大对职业教育的扶持力度，重点扶持26所市级以上骨干职业学校发展。进一步抓好泉州师院规划建设，继续支持办好华侨大学、仰恩大学、黎明大学、泉州电大等本、专科院校，提高现有高专、高职院校的办学水平。继续扶持和依法规范民办学校办学。至年底争取80％以上的中小学生接受信息技术教育。加强学前教育、成人教育、老年教育和特殊教育。强化人才培养。落实进一步加强人才工作措施，创新人才激励机制，吸引更多优秀人才到我市创业发展。继续实施“112专家工程”、“222高等技能人才培养工程”。加强卫生事业。加大对卫生事业的投入，启动市第一医院新院和泉州医疗中心（一期）建设，着手规划建设福建医大附属二院新院。认真落实各项疾病预防控制措施，重点抓好高致病性禽流感、艾滋病等重大传染病防治。完善城乡卫生服务体系，推进县级中医院、妇幼保健院和12所乡镇卫生院标准化建设，扶持6个社区服务中心和253个“空白村”设立卫生所，建立乡村医生津贴补助和乡镇医技人员培训制度。积极探索建立多种形式的农民健康保障体系，新增德化、晋江等县（市）为新型农村合作医疗试点。切实加强卫生行风建设，整顿医疗服务市场。发展文化事业。建好用好中国闽台缘博物馆，办好海峡西岸论坛。继续做好“海上丝路”申报“世遗”和泉州南音申报“人类口头及非物质遗产代表作”工作。认真实施中国民族民间文化保护工程综合性试点城市工作，重视“泉州学”研究，整理、编纂、出版《泉州文库》、《泉州南音集成》丛书。建设梨园古典剧院、南音艺苑等社会事业项目。推进创建文化先进县活动，发展文化产业。加大“扫黄打非”力度，加强文化市场管理。启动数字电视工程。开展全民健身活动，组队参加第十三届省运会，备战2008年第六届全国农民运动会。加快发展社会科学、文学艺术、档案、气象、地方志等社会事业。加强精神文明建设。深化“道德新风与你同行”、“十百千万工程”等活动，强化公共道德宣传，积极推进文明城市、文明村镇、文明行业、文明社区、文明单位创建活动；加强和改进未成年人思想道德建设；深入实施泉州市妇女儿童发展纲要；加强国防教育与国防动员，做好民兵、预备役工作，落实各项优抚政策，妥善安置退伍、转业军人和随军家属，积极支持部队建设，争创全国双拥模范城“六连冠”。

　　（六）加快推进和谐社会建设，开创社会和谐平安新局面

　　妥善处理不同利益群体关系。建立健全社会矛盾化解机制，大力推进和谐社区、和谐村镇、和谐单位建设，倡导人与人和睦相处。贯彻《宗教事务条例》，做好民族与宗教工作。认真落实新《信访条例》，提高信访工作法制化水平。促进就业和再就业。进一步解决我市用工结构性矛盾，完善积极的就业政策，重点做好大中专毕业生、城镇新增劳动力和农村劳动力就业工作，全年新增就业14万人，城镇登记失业率控制在1.9％以下。加强劳动力培训，实现培训各类劳动力25万人、转移农村劳动力5万人目标。完善社会保障体系。扩大社会保障覆盖范围，参加基本养老、失业、医疗、工伤和女职工生育保险人数分别达到41万、41万、47万、33万和29万人。做好企业离退休人员养老保险待遇调整工作，完善机关事业单位养老保险制度。稳妥推进城镇居民、被征地农民参保扩面工作，逐步解决进城务工人员社会保障问题，完善城乡居民最低生活保障制度。加强社会福利事业建设，提高“五老”人员、“五保户”和残疾人、孤残儿童等特殊困难群体的福利服务和保障水平。推进“平安泉州”建设。完善社会治安综合治理长效机制，健全防控体系，妥善处理群体性事件，严厉打击有组织犯罪、严重暴力犯罪和多发性侵财犯罪，开展人民禁毒战争。落实安全生产责任制，有效遏制重特大事故发生。

　　（七）加快推进生态市建设，建设资源节约型和环境友好型社会

　　坚持节约优先。抓紧制定泉州市节能工程实施方案，抓好石油化工、纺织、建材等重点耗能行业和年能耗3000吨标准煤以上企业的节能工作；推动交通运输、住宅与公共建筑物、城市生活和公共聚集场所节能；开发利用太阳能和地热能源。抓紧出台《泉州市节约用水管理暂行规定》，认真实施总量控制、定额管理的计划用水制度。规范矿业开发秩序，推进矿产集约化、规模化开采经营。节约和集约使用海域、岸线。推进综合利用。实施循环经济发展规划，积极推行清洁生产和ISO14000环境管理体系认证，继续抓好循环经济示范项目。推进工业废物资源化综合利用及旧家电、车辆等废弃物集中处理和回收再生利用。完成土地利用总体规划修编，实施土地整理，加强土地利用管理，严格耕地保护制度，盘活城乡闲置土地，建设节地型工业园区。促进可持续发展。坚持计划生育基本国策，稳定低生育水平，提高人口素质，有效治理出生人口性别比偏高问题，加大流动人口计生管理服务力度，人口自然增长率控制在8.5‰以内。强化环境治理，制订实施“十一五”环保目标责任书和“创模”持续改进计划，完成“两江”流域年度环境保护与控污任务。争取各县（市、区）垃圾无害化处理、污水处理设施建成投入运行，晋江流域省控断面水质达标率90％以上，中心市区饮用水源水质达标率97％以上。继续加强对国道、省道及晋江、洛阳江沿岸“青山挂白”和近海、泉州湾海域环境污染综合整治。抓好城市烟、粉、尘、固体废弃物、机动车尾气和噪声污染控制。实施生态公益林保护、沿海防护林、生物多样性、绿色通道和城乡绿化、森林防火减灾五大工程。开展生态示范乡（镇）创建活动。加强地震监测跟踪，完善气象灾害、地质灾害、海洋灾害等防灾减灾系统，建设生态型宜居城市。

　　四、加强政府建设，确保“十一五”目标任务圆满完成

　　牢记宗旨，进一步提高为民服务水平。坚持为民惠民利民，认真落实2006年市委、市政府12件25项为民办实事项目，取信于民。努力解决群众关心的看病难、环境污染、食品安全等问题。创新服务方式，推行集中审批和网上审批，深化电子政务建设，加强政务公开，增强工作透明度。健全社会公示、社会听证制度，让人民群众更广泛地参与公共事务管理。加强各种自然灾害、公共突发性事件的预测预报，健全预警体系，提高公共安全管理和应急处置能力，保证人民群众生命财产安全。

　　转变职能，进一步完善行政管理体制。认真贯彻建设公共服务型政府的意见，抓好经济调节和市场监管，加强社会管理和公共服务。健全科学民主依法决策机制，完善行政决策监督和责任追究制度。充分发挥城市综合执法机构作用，强化城市管理。积极稳妥推进事业单位改革，依法规范对事业单位的授权行为。推进机构和编制的法定化。整顿和规范市场经济秩序，重点打击制假售假、商业欺诈、不正当竞争、偷税骗税和侵犯知识产权行为。加快建设社会信用体系，健全失信惩戒制度。

　　依法行政，进一步推进法治政府建设。加强民主法制建设，制定实施“五五”普法规划，全面完成依法治市中期目标，不断提高人民群众法律素质和公务人员依法办事水平。认真贯彻《全面推进依法行政实施纲要》，建设法治型、责任型政府。深化行政执法体制改革，推行行政执法责任制和行政问责制，坚决纠正和查处执法不公和行政不作为、乱作为问题。

　　从严治政，进一步树立高效廉洁形象。牢记“两个务必”，大兴求真务实之风，坚持用科学发展观指导工作、判断得失，力戒心浮气躁，防止急于求成，切实把经济社会发展的各项工作落到实处。加强机关效能建设，认真落实政府绩效考评和“日考勤、季评议、年述职、定期考核”等制度。落实党风廉政建设责任制，进一步完善教育、制度、监督并重的惩治和预防腐败体系，坚决纠正损害群众利益的不正之风。加强审计工作和行政监察，从源头上防止腐败。组织实施《公务员法》，加强公务员理论学习、业务培训和能力建设，努力建设人民满意的公务员队伍。

　　时代赋予我们重任，人民寄予我们厚望。我们要在市委领导下，自觉接受市人大及其常委会监督，完善向人大及其常委会报告工作的制度，认真执行人大及其常委会的决议、决定，积极支持政协履行政治协商、民主监督和参政议政职能，认真听取各民主党派、工商联、无党派人士以及专家学者的意见，及时办理人大代表议案、建议和政协提案，发挥工会、共青团、妇联等群团组织作用，共同推进我市改革和发展大业。

　　各位代表！海峡西岸正春风，宏伟蓝图催人进。让我们紧密团结在以胡锦涛同志为总书记的党中央周围，高举邓小平理论和“三个代表”重要思想伟大旗帜，全面落实科学发展观，在市委领导下，紧紧依靠全市人民，同心同德，奋发图强，为全面完成2006年工作任务，为实现“十一五”规划和全面建设小康社会而努力奋斗！
